# Physical Passaging of Embryoid Bodies Generated from Human Pluripotent Stem Cells

**DOI:** 10.1371/journal.pone.0019134

**Published:** 2011-05-03

**Authors:** Mi-Young Son, Hyun-jin Kim, Min-Jeong Kim, Yee Sook Cho

**Affiliations:** 1 Development and Differentiation Research Center, Korea Research Institute of Bioscience and Biotechnology, Yuseong-gu, Daejeon, Republic of Korea; 2 Department of Functional Genomics, University of Science and Technology, Yuseong-gu, Daejeon, Republic of Korea; Wellcome Trust Centre for Stem Cell Research, United Kingdom

## Abstract

Spherical three-dimensional cell aggregates called embryoid bodies (EBs), have been widely used in *in vitro* differentiation protocols for human pluripotent stem cells including human embryonic stem cells (hESCs) and human induced pluripotent stem cells (hiPSCs). Recent studies highlight the new devices and techniques for hEB formation and expansion, but are not involved in the passaging or subculture process. Here, we provide evidence that a simple periodic passaging markedly improved hEB culture condition and thus allowed the size-controlled, mass production of human embryoid bodies (hEBs) derived from both hESCs and hiPSCs. hEBs maintained in prolonged suspension culture without passaging (>2 weeks) showed a progressive decrease in the cell growth and proliferation and increase in the apoptosis compared to 7-day-old hEBs. However, when serially passaged in suspension, hEB cell populations were significantly increased in number while maintaining the normal rates of cell proliferation and apoptosis and the differentiation potential. Uniform-sized hEBs produced by manual passaging using a 1∶4 split ratio have been successfully maintained for over 20 continuous passages. The passaging culture method of hEBs, which is simple, readily expandable, and reproducible, could be a powerful tool for improving a robust and scalable *in vitro* differentiation system of human pluripotent stem cells.

## Introduction

The unlimited potential of human embryonic stem cells (hESCs) and human induced pluripotent stem cells (hiPSCs) to differentiate into all of the cell types present in the body provides great opportunities for basic research, drug discovery, and regenerative medicine. However, a major bottleneck to the ultimate use of these cells is the large-scale production of pluripotent hESCs/hiPSCs and their differentiated derivatives. Substantial progress has been achieved with respect to culture conditions that support the long-term and scalable expansion of hESCs/hiPSCs [1]–[4], but current techniques still do not allow efficient, economized large-scale production.

To derive or obtain tissue-specific differentiated cells from hESCs/hiPSCs, most *in vitro* hESC/hiPSC differentiation protocols requires an initial spontaneous formation of embryoid bodies (EBs), which is a common and critical intermediate for the induction of lineage-specific differentiation [5]–[7]. Conventionally, human embryoid bodies (hEBs) can be formed in suspension cultures through releasing attached cultured hESC/hiPSC colonies to culture medium using enzymatic and mechanical (scraping) methods and further being maintained in suspension without subculture. An early stage of hEBs maintained for about 10 days in suspension were largely used for further differentiation into specific lineage. However, such techniques yield a heterogeneous cell population both between and within aggregates, which results in a limited developmental potential and a low production yield. Report showed that long-term culturing of hEBs could be achieved under the physiological media glucose condition while maintaining embryonic developmental potential, but in a chaotic and disorganized way [8]. Therefore, alternative methods to facilitate hEB formation and culture systems have been explored, including stirred suspension cultures [9], [10], bioreactor cultures [11], and the formation of spin EBs that are aggregated by centrifugation [12], [13]. Study also showed that production of hEBs with a more uniform size could be advanced using the single-cell suspension method and the 3-D cuboidal microwell system [14]. But still lack of knowledge of specific phenotypic features and a method of large-scale expansion and long-term propagation of hEBs limits the usefulness of human pluripotent stem cells.

In this paper, we have established and characterized a subculture method of hEBs that allows the scalable and the long-term propagation of hEB without loss of their proliferation and differentiation potential. This method, when combined with suspension culture system with bioreactors, will facilitate the large-scale expansion of hEBs and hEB-based differentiation for cell-based therapeutics.

Our culture method of hEBs achieved in this study may substantially contribute to bypass or diminish the requirement for the large-scale production of hESCs/hiPSCs and to advance and ensure the quality of hEBs, such as consistent morphological and developmental characteristics (*i.e.*, size, shape, and multi-lineage differentiation potential).

## Materials and Methods

### hESC and hiPSC cultures

The human embryonic stem cell (hESC) lines H9 (NIH Code, WA09, XX, passages 28–47; WiCell Research Institute, Madison, WI) and H1 (NIH Code, WA01 XY, passages 33–40; WiCell Research Institute) and human induced pluripotent cell (hiPSC) lines [15] were routinely maintained on γ-irradiated mouse embryonic fibroblasts (MEFs) in hESC culture medium consisting of 80% DMEM/F12 medium, 20% knockout serum replacement (KSR, Invitrogen), 1% non-essential amino acids (NEAA, Invitrogen), 1 mM L-glutamine (Invitrogen), 0.1 mM β-mercaptoethanol (Sigma, St. Louis, MO) and 6 ng/ml basic fibroblast growth factor (bFGF, Invitrogen). MEFs were prepared from day 12.5 p.c. fetuses of CF-1 mice according to standard procedures [16]. The cells were passaged using mechanical or collagenase-based enzymatic methods every 5–6 days [17]. All cultures were routinely screened for mycoplasma, and normal karyotype was monitored by chromosomal G-band analysis (GenDix, Inc., Seoul, Korea). Our research was performed under ethical approval from the Institutional Review Board (IRB) at KRIBB, Korea (KRIBB-IRB-20090916-1).

### hEB propagation

**Figure 1:**
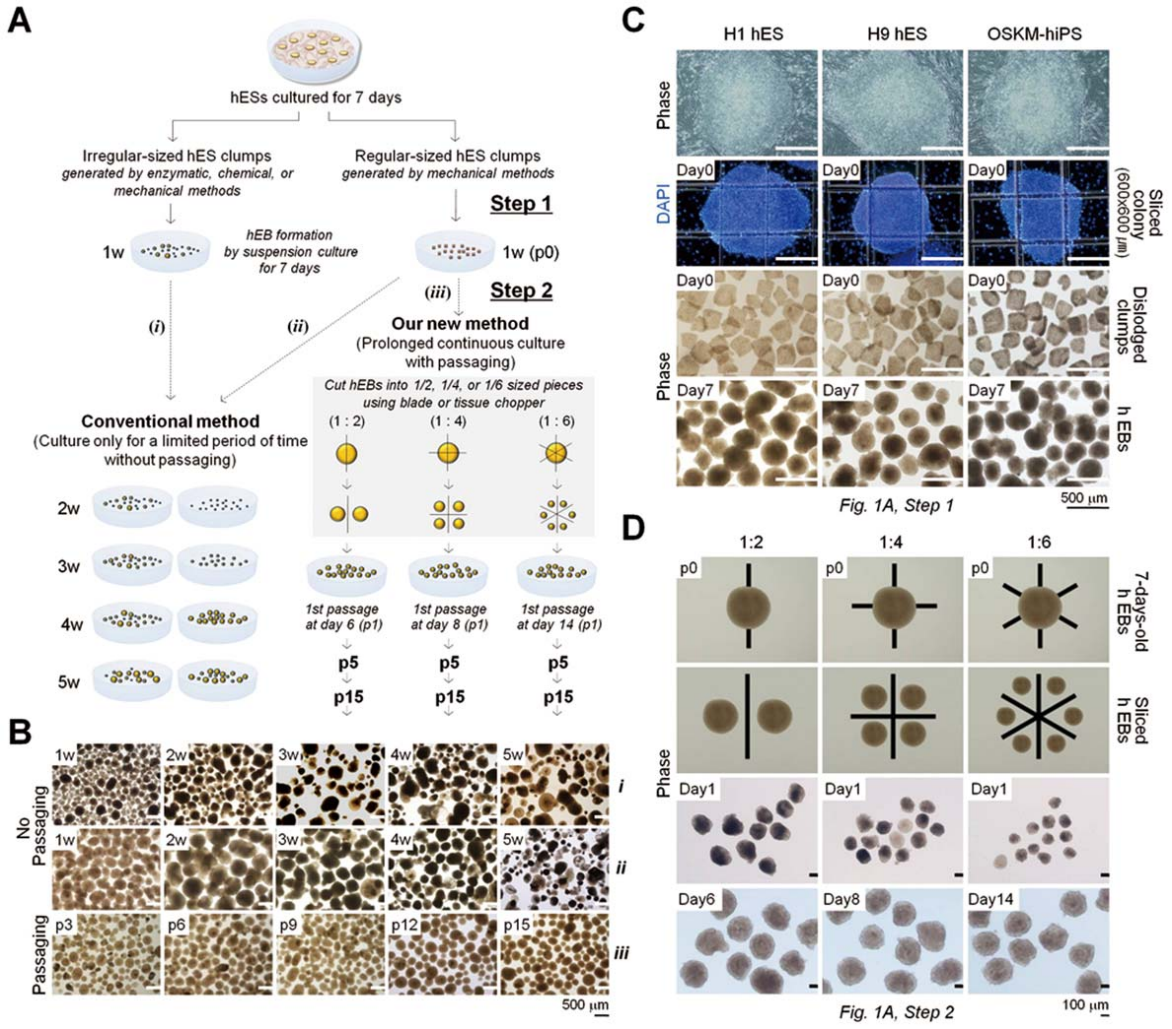
Long-term-cultured hEBs derived from hESCs and hiPSCs. (A) Schematic representations of the hEB culture conditions with and without passaging. w: week, p: passage. Step 1: Period of hEB formation, Step 2: Period of hEB maintenance and expansion. (B) Morphologies of the cultured hEBs with or without passaging for various times as indicated. (*i* and *ii*) H9 hESC-derived hEBs cultured without passaging (conventional method). (iii) H9 hESC-derived hEBs cultured with passaging. For subculturing, hEBs were sliced at a ratio of 1∶4 every 7 days. The medium was changed every 3 days (i–iii). (C) Representative images of regular-sized hEB formation. (*Top*) Seven-day-old (7 d) hESCs (H1 and H9 hES) and OSKM-induced hiPSCs (OSKM-hiPS). (*Middle top*) hESC and iPSC colonies sliced into a grid pattern (600×600 µm) using a McIlwain tissue chopper prior to dislodgement. Cell nuclei were visualized with DAPI. (*Middle bottom*) Mechanically dislodged colonies in suspension culture. (*Bottom*) The 7 d-hEB population in suspension culture. (D) Representative images of the hEB passaging process. The 7 d-hEBs derived from H9 hESCs (*Top*) were mechanically dissociated into smaller aggregates at ratios of 1∶2, 1∶4, and 1∶6 (*Middle top*). (*Middle bottom*) Phase contrast images of passaged hEBs cultured for 1 day. (*Bottom*) Representative images of passaged hEBs exhibiting complete re-growth to normal size.

For hEB maintenance, three different methods were used, as depicted in Figure 1a. In the first method (Fig. 1A*i*), 7-day-old hESCs/hiPSCs were harvested with collagenase IV (1 mg/ml) and dispersed into small clumps by scraping and pipetting. The resulting clumps were added to the non-tissue culture treated plastic petri dish and cultured in suspension for the indicated periods in hEB medium consisting of Knockout DMEM (Invitrogen), 20% FBS (Invitrogen), 1% non-essential amino acids, 1 mM L-glutamine, 0.1 mM β-mercaptoethanol, and 1% penicillin/streptomycin (Fig. 1A*i*). Seven-day-old hESCs/hiPSCs were disaggregated into regular-sized squares (600×600 µm) using a McIlwain tissue chopper to produce uniform hESC clumps (Fig. 1A*ii* and *iii*). In the second method (Fig. 1A*ii*), uniform clumps were seeded onto plastic petri dishes and cultured continuously in suspension for the indicated periods in hEB medium without subculture. In the third method proposed herein (Fig. 1A*iii*), uniform clumps that were seeded onto plastic petri dishes were cultured in suspension for 7 days and periodically passaged after dissociation at various ratios (1∶2, 1∶4, and 1∶6) on the indicated days (Fig. 1A*iii*). The third method for hEB passaging was done by draining away the medium to fasten the EBs to the dish, then the EBs were cut by a McIlwain tissue chopper or sterile razor blade. Fully grown hEBs were sliced at a ratio of 1∶4 for subculturing and for further studies unless indicated otherwise. The culture medium was refreshed every 2 days.

### hEB growth analysis

The average diameter of the hEB populations was calculated by measuring the large and small diagonals of ten representative hEBs in each experimental group. To count the total number of cells within each EB, the hEBs were dissociated into single-cell suspensions with 0.1% trypsin-EDTA for 3 min. Live cell numbers were determined by trypan blue (Invitrogen) exclusion. For the cell counts, three to ten hEBs were counted in each experimental group.

### Spontaneous in vitro differentiation of hEBs

After five days of growth in suspension, the cell aggregates were seeded onto gelatin-coated plates and cultured in hEB medium for an additional 10 days. The medium was changed every two days.

### In vitro differentiation of hEBs into specific lineages

To derive neural stem spheres (NSSs), the hEBs were cultured in NSS culture medium (DMEM/F12 supplemented with 1× N2/B27 (Invitrogen), 20 ng/ml epidermal growth factor (Invitrogen), 20 ng/ml bFGF, 10 ng/ml leukemia inhibitory factor (Sigma) and 100 U/ml penicillin-streptomycin). The NSSs were sub-cultured every week using a McIlwain tissue chopper (Mickle Engineering, Gomshall, Surrey, UK), and the medium was refreshed every 2 days. For terminal differentiation into neurons, oligodendrocytes, astrocytes, the NSSs were allowed to attach to Matrigel-coated coverslips and maintained in the same NSS medium without growth factors for 3–4 weeks. For osteoblast differentiation, the hEBs were plated in Matrigel-coated dishes and cultured in medium containing osteogenic supplements and 0.1 mM L-ascorbic acid (Sigma), 10 mM β-glycerophosphate (Sigma), and 0.1 mM dexamethasone (Sigma) for 3–4 weeks. For cardiomyocyte differentiation, the hEBs were plated in gelatin-coated cell culture dishes in differentiation medium consisting of Knockout DMEM (Invitrogen), 20% FBS (Invitrogen), 1% non-essential amino acids, 1 mM L-glutamine, 0.1 mM β-mercaptoethanol, and 1% penicillin/streptomycin for 3 weeks. For endothelial cell differentiation, the following protocol was used: hEBs were maintained in hEB medium supplemented with 20 ng/ml BMP4 (R&D Systems) (removed on day 7); on day 1, the medium was supplemented with 10 ng/ml activin A (Peprotech) (removed on day 4); on day 2, the medium was supplemented with 8 ng/ml bFGF (R&D Systems) (for the duration of the culture); on day 4, the EBs were transferred to adherent conditions in Matrigel-coated plates, and the medium was supplemented with 25 ng/ml VEGF-A (Peprotech) (for the duration of the culture); finally, on day 7, 10 µM SB431542 (Sigma) was added to the culture followed by an incubation for 7 days.

### PCR analysis

Total RNA was isolated from the cells using the RNeasy Mini Kit (Qiagen, Valencia, CA) and reverse-transcribed using the SuperScript First-strand Synthesis System Kit (Invitrogen) according to the manufacturers' protocols. Semi-quantitative RT-PCR and real-time PCR were performed using methods and primers described in Text S1 and Table S1.

### Immunocytochemistry

Cells that had been cultured on gelatin-coated 4-well Lab-Tek chamber slides (Nunc, Naperville, IL) were fixed with 4% paraformaldehyde, permeabilized in PBS/0.2% BSA/0.1% Triton X-100 and blocked with 4% normal donkey serum (Molecular Probes, Eugene, OR, USA) in PBS/0.2% BSA for 1 h at RT. After blocking, the cells were incubated with the respective primary antibodies diluted in PBS/0.2% BSA. After washing, the cells were incubated with FITC- or Alexa 594-conjugated secondary antibodies (Invitrogen) in PBS/0.2% BSA for 1 hr at RT. Nuclei were then counterstained with 10 µg/ml DAPI (4′,6-diamidino-2-phenylindole). The chamber slides were analyzed using an Olympus microscope or an Axiovert 200 M microscope (Carl Zeiss, Gottingen, Germany). The antibodies used are listed in Table S2.

## Results and Discussion

**Figure 2:**
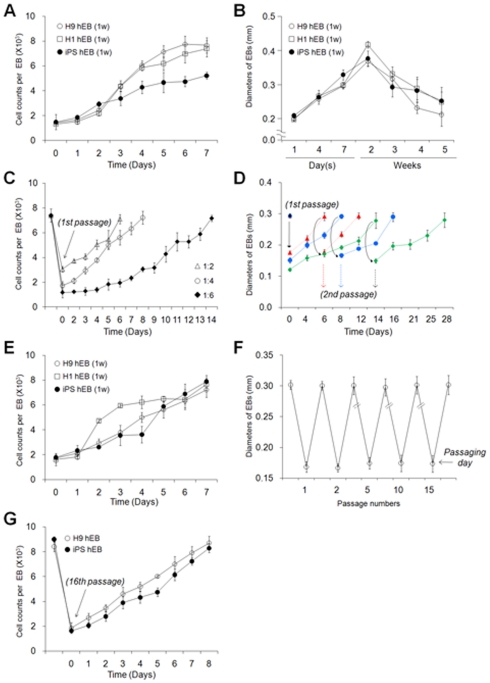
Size and number of hEBs under various culture conditions. The growth curves were determined either by counting the average number of cells per hEB or by measuring the mean diameter of each hEB. (A) hEB cell counts during the first week of incubation. Step 1 culture shown in *Fig. 1A*. (B) Diameters of the hEBs during long-term culture without passaging. Step 1(1 to 7 days) and Step 2 (1 to 5 weeks) cultures shown in *Fig. 1A*. (C) Cell counts for the passaged H9 hEBs. (D) Diameters of the passaged H9 hEBs. hEBs were passaged at ratios of 1∶2, 1∶4, and 1∶6 (C and D). (E) Cell counts for hEBs that were passaged five times. hEBs were passaged at a ratio of 1∶4. (F) Diameters of the H9 hEBs during 15 continuous serial passages. hEBs were passaged at a ratio of 1∶4. The results represent the mean values (± SEM) of samples obtained from a representative experiment performed in triplicate. w: week, p: passage. Arrows indicate the passaging day (C, D, and F). (G) Cell counts for hEBs that were passaged 16 times.

In general, after the formation of hEBs in suspension for 2–4 days, they can be further maintained in culture for approximately one month with renewal of the medium every 2–3 days without passaging (Fig. 1A*i* and *ii*). During cultivation, an increase in the cell number and size was observed in hEB cell populations up to about 2 weeks (Fig. 1B*i* and *ii*, Fig. 2A and B, and Table S3). However, after a prolonged incubation (over 2 weeks), a decrease in healthy normal hEBs, an increase in hEB heterogeneity and cell debris, and vascularized cystic hEBs were all observed, despite the initial formation of uniform hEBs (Fig. 1B*i* and *ii* and Fig. 2B). These morphological characteristics are suggestive of both decreased cell growth and proliferation and increased apoptosis in long-term-cultured hEB populations. Under these conditions, the ability to increase hEB production requires scaling-up of the hESC/hiPSC culture process, which involves variegated, time-consuming, and expensive methods and large quantities of hESC clumps.

Therefore, we attempted to develop a simple, efficient method for scaling-up hEB production through hEB passaging as illustrated in Figure 1A*iii*. The hiPSCs used in the present study were generated from human fibroblasts through retroviral transduction of the four transcription factors Oct4, Sox2, Klf4, and c-Myc (Fig. S1). First, we acquired regular-sized 7-day-old hEBs (mean diameter of 290 µm, 7.6×10^3^±0.61 cells per hEB) from hESC/hiPSC clumps (600×600 nm, 1.7×10^3^±0.187 cells per clump) (Tables S3 and S4). These regular-sized clumps were prepared from monolayer-cultured hESCs/hiPSCs using automated tissue choppers (Fig. 1C). Next, 7-day-old hEBs were passaged by cutting them using sterile razor blades into 2, 4, or 6 pieces which were almost the same shape and size, and then they were maintained in a suspension culture (Fig. 1A*iii* and D). Passaged hEBs demonstrated successful re-growth back to volumes and cell numbers comparable to those observed for 7-day-old hEBs. These results were observed after 6 (2 pieces, 1∶2 ratio), 8 (4 pieces, 1∶4 ratio), and 14 (6 pieces, 1∶6 ratio) days of incubation, as confirmed by a morphological observation (Fig. 1B and D), diameter measurements (Fig. 2D and F) and cell counts (Fig. 2C and E). Size-controlled hEBs (600×600 mm) derived from hESCs and hiPSCs were periodically passaged by mechanical splitting (1∶4 ratio) and successfully maintained for more than 15 passages (Fig. 1B*iii*, Fig. 2F and G).

**Figure 3:**
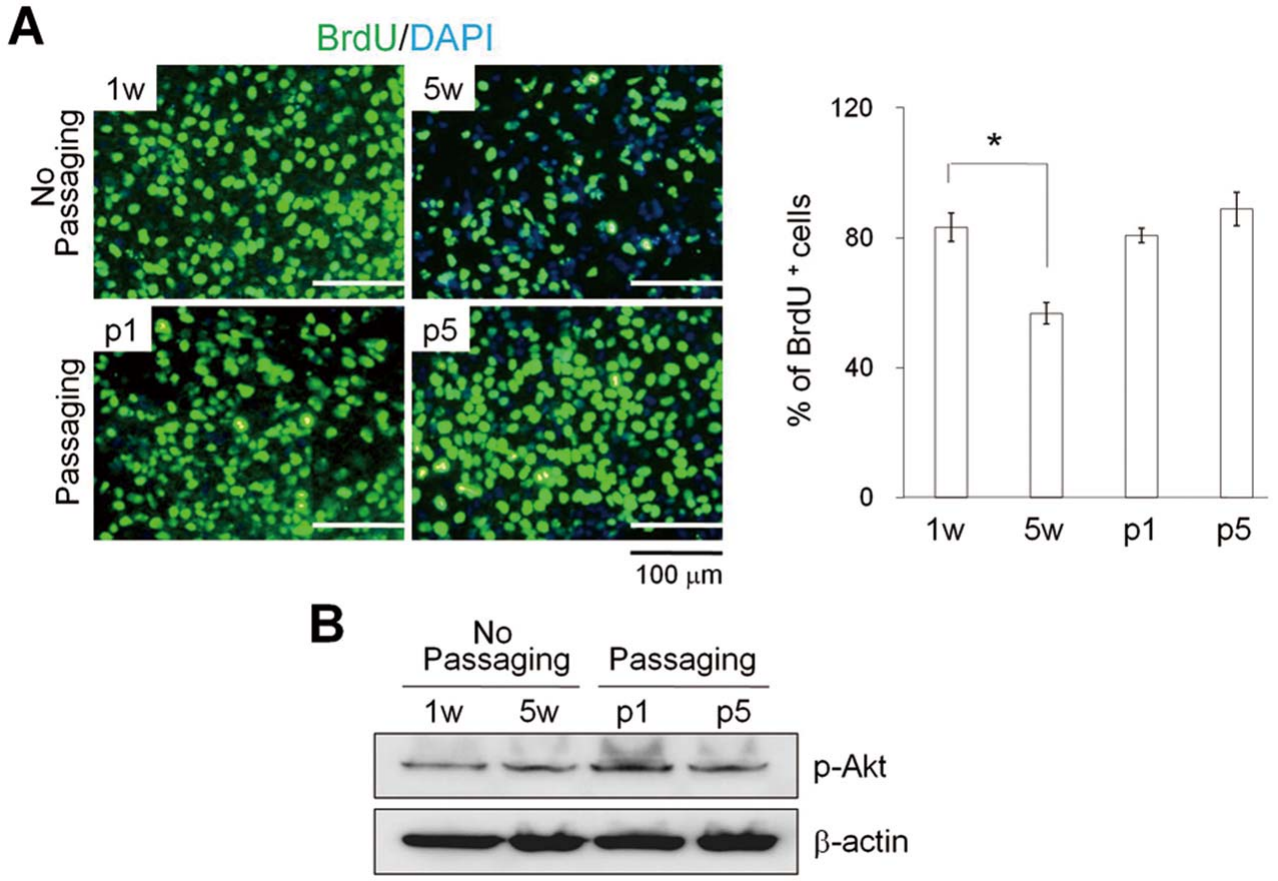
Proliferation rates for hEBs. The 7 d-hEBs (1 w) were cultured without passaging for 5 weeks (5 w) and with passaging (p1 and p5). (A) Representative images (*left panel*) and quantification of BrdU^+^ cells (*right panel*). The relative number of BrdU^+^ cells per field of vision was quantified, and the results are presented as the percentage of the total number of cells counted. The data represent the mean ± SEM (n = 3). (B) Western blot analysis of phosphorylated Akt proteins. w: week, p: passage.

Passaged hEBs (passage 1, 2, 5, or 15) showed no marked differences in cell growth (Fig. 2C, D, and F) or the proliferation rate (Fig. 3A) when compared to freshly formed 7-day-old hEBs (7 d-hEBs). In contrast, hEBs maintained under un-passaged conditions for 5 weeks (5 w-hEBs; 56.7±3.2%) displayed a marked decrease in cell proliferation as compared to 7 d-hEBs (83.2±4.4%) and passaged hEBs (hEBs at passage 1; 80.7±2.2%, hEBs at passage 5; 85.1±5.2%), as determined by BrDU^+^ cell counts (Fig. 3A). However, the decrease in hEB cell proliferation was not associated with the activity of the multifunctional serine/threonine kinase Akt, which plays a critical role in signal transduction pathways involved in cell proliferation (Fig. 3B) [18]. These results suggest that un-passaged hEBs were less proliferative compared to passaged hEB cell populations.

**Figure 4:**
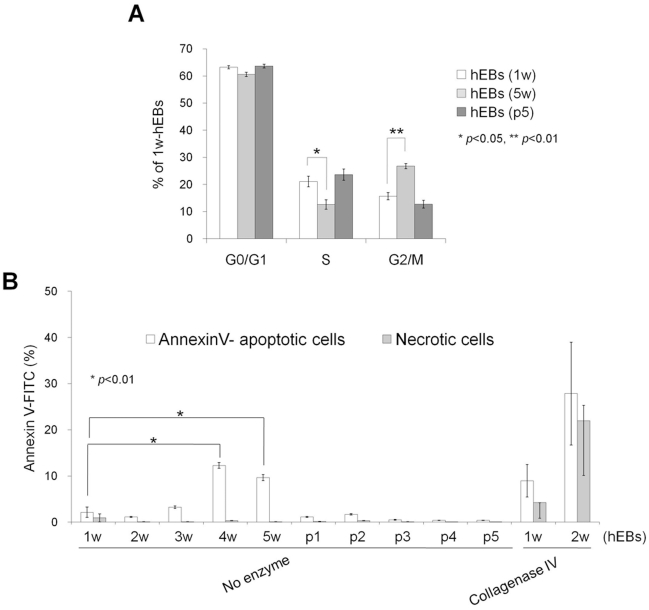
Cell-cycle and apoptosis analysis of hEBs. (A) Cell-cycle analysis of hEBs using propidium iodide staining. The percentages of cells in G1, S, and G2/M are depicted in the graph representing the means ± SEM (n = 3). (B) Apoptosis analysis of hEBs using annexin-V/PI staining. hEBs were formed either with or without enzymatic treatment (collagenase IV) and maintained either with (p1, p2, p3, p4, and p5) or without passaging (1 w, 2 w, 3 w, 4 w, and 5 w). Values shown in the graphs represent the mean ± SEM (n = 3).

**Figure 5:**
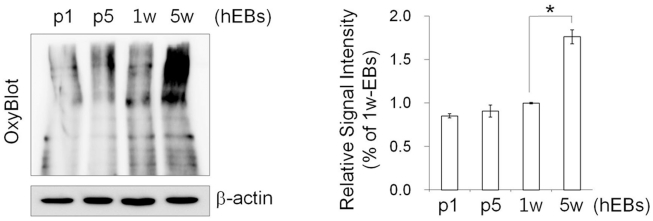
Protein oxidation assay in hEBs. *Left*: Representative image for the OxyBlot assay, which detects the level of proteins with oxidative modifications. *Right*: For protein quantification, the blots were scanned, and the bands were quantified by densitometry. The data represent the mean ± SEM (n = 3).

Next, we characterized the effect of hEB passaging on cell cycle and apoptosis. Flow cytometry-based cell-cycle analysis showed that a large number of hEB cell populations contained a relatively high percentage of G0/G1 phase cells (63.23%±0.59) and a low percentage of S (21.1%±1.92) and G2/M phase cells (15.68%±2.33) in 7 d-hEBs (Fig. 4A). Passaged hEBs displayed no marked differences in the cell-cycle distribution relative to 7 d-hEBs, however, 5 w-hEBs demonstrated a reduced number of cells in S phase (12.64%; p<0.05) and an increased number of cells in G2/M phase (26.78%; p<0.01) (Fig. 4A). In parallel, passaged hEBs displayed no apparent signs of apoptosis, as indicated by the lack of a significant induction of annexin-V-binding populations (Fig. 4B). However, an approximately 6-fold increase in annexin-V-positive apoptotic cells was observed in hEBs that were maintained under un-passaged conditions for 4 weeks (12.30%; p<0.01) as compared to 7 d-hEBs (2.16±1.12%). By contrast, passaged hEBs (passage 5; 0.41±0.04%) demonstrated fewer apoptotic cells (Fig. 4B). As expected, an increase in apoptosis was observed in hEBs that were generated using collagenase IV treatment compared to those that were not treated with the enzyme, and annexin-V-positive cells were evident within 1–2 weeks of the incubation (Fig. 4B). Reports have shown that the levels of mitochondrial biosynthesis, DNA damage, and oxidative stress are up-regulated while telomerase activity is down-regulated during hESC differentiation [19], and hiPSCs possess stress defense mechanisms that are similar to those of hESCs [20]. As expected, the carbonyl signal was significantly increased by approximately 1.76-fold in 5 w-hEBs cultured without passaging compared to 7 d-hEBs, whereas in passaged hEBs (passages 1 and 5), this signal remained unchanged (Fig. 5). These data suggest that increased oxidative stress may contribute to the increased apoptosis observed in long-term-cultured hEBs under un-passaged conditions.

**Figure 6:**
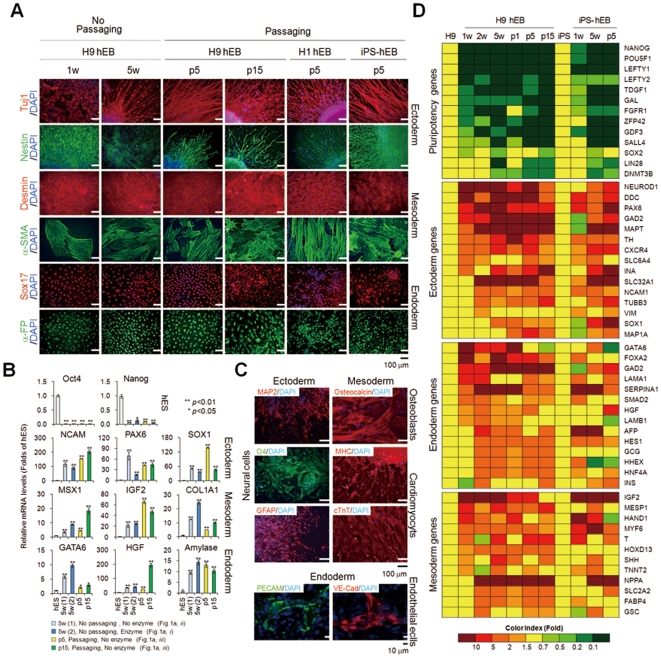
*In vitro* differentiation of hEBs. (A) Immunocytochemical analysis of the spontaneous differentiation of passaged hEBs. Lineage-specific markers of three germ layers, ectoderm (Tuj1 and nestin), mesoderm [desmin and α-smooth muscle actin (α-SMA)], and endoderm [Sox17 and α-fetoprotein (AFP)] were detected in hEBs derived from hESCs (H9 and H1) and hiPSCs cultured with or without passaging. Nuclei were stained with DAPI. H9 hEB; hEBs derived from H9 hESCs, H1 hEB; hEBs derived from H1 hESCs, iPS-hEBs; hEBs derived from hiPSCs. (B) Quantitative real-time PCR analysis of spontaneous differentiation using markers of pluripotency (Oct4 and Nanog) and the three germ layers [ectoderm (NCAM, PAX6, and SOX1), mesoderm (MSX1, IGF2, and COL1A1), and endoderm (GATA6, HGF, and amylase)] in H9 hEBs cultured with or without passaging. For hEB formation, hESCs were dislodged either in the presence or absence of enzymatic treatment (collagenase IV). Each group was compared with control hESCs. Statistical significance was calculated using Student's t-test: *P<0.05; **P<0.01 (C) *In vitro* differentiation of passaged H9 EBs into specific lineages, including neural cells (ectoderm), osteoblasts (mesoderm), cardiomyocytes (mesoderm) and endothelial cells (endoderm). *Ectoderm*: Neural stem spheres derived from hEBs (passage 17) were allowed to attach to culture dishes and further differentiated into mature neural phenotypes. Neural cells were detected by positive immunostaining for neuronal markers: MAP2 (neurons), O4 (oligodendrocytes) and GFAP (astrocytes). *Mesoderm*: Osteoblasts differentiated from hEBs (passage p15) were characterized by positive staining for mineralized bone nodule-specific alizarin red S and osteocalcin proteins. Cardiomyocytes differentiated from hEBs (passage p15) were characterized by positive immunostaining for cTnT, MHC, and Nkx2.5. *Endoderm*: Endothelial cells differentiated from hEBs (passage p15) were characterized by positive immunostaining for PECAM and VE-cadherin (VE-cad). Cell nuclei were visualized with DAPI. (D) Heat map analysis of pluripotency and lineage-specific marker genes. A global gene expression analysis in H9 hESCs (H9), OSKM-hiPSCs (iPS), H9 hEBs, and iPS-hEB was conducted using Agilent Human Genome 44k Arrays. Pluripotency and lineage-specific genes were selected, and their expression levels in hEBs and hESCs/hiPSCs were analyzed. Mean gene expression ratios of hEBs relative to hESCs or hiPSCs were calculated and the ratios are color-coded, as indicated by the color index bar. Red: up-regulated genes as compared to control cells (hESC/hiPSC), green: down-regulated genes as compared to control cells (hESC/hiPSC), yellow: control results determined for hESCs and hiPSCs were set at a value of 1.

To demonstrate the differentiation potential of passaged hEBs, we examined the differentiation capacity of passaged EBs derived from hESCs and hiPSCs *in vitro*. We observed that hEBs maintained both with and without subculture were fully differentiated, as evidenced by the down-regulation of pluripotency markers and up-regulation of mRNAs (Fig. 6B and Fig. S2A and B) and/or proteins (Fig. 6A) specific for ectoderm, mesoderm, and endoderm markers. Statistical analysis of multiple markers (NCAM, PAX6, IGF2, COL1A1, GATA6, HGF, and Amylase) for three-germ layers showed that linage-specific maker genes displayed relatively constant expression levels among hEBs of different passages, whereas some markers such as SOX1, MSX1, and HGF displayed different expression levels (Fig. 6B). Developmental potential of long-term-cultured hEBs (≥15 passages) was further confirmed by their successful differentiation into specific lineages, such as neural cells (neurons, oligodendrocytes, and astrocytes), osteoblasts, cardiomyocytes, and endothelial cells by confirming the expression of markers specific for each lineage at the mRNA (Fig. S2C) and protein level (Fig. 6C). Although some slight differences were observed among the hEB populations, a microarray analysis using Agilent's Whole Genome Human 44K also confirmed the common up-regulation of selected lineage marker genes for ectoderm, mesoderm, and endoderm and the concomitant down-regulation of pluripotency marker genes in hEBs maintained both with and without subculture (Fig. 6D and Table S3). In addition, the long-term-maintained hEBs with continuous passaging retained normal karyotypes (Fig. S3). These results indicate that the regenerative abilities are maintained in EBs during propagation by serial passage. However, more detailed further studies are needed to determine whether trivial differences on lineage-specific gene expression observed in EBs of different passages can influence (thereby limits) the differentiation potential of EBs.

Overall, the long-term-maintained hEBs (over 2 weeks) without subculture exhibited a decrease in cell proliferation, G2/M arrest, an increased level of apoptosis, and free radical protein damage. These biological and physical obstacles raised during long-term culture of hEBs can be overcome with our new culture method that utilizes the passaging of hEBs. hEBs maintained for long-term (≥15 passages) are proliferative, show low rates of apoptosis, and possess a multilineage differentiation potential similar to that observed in freshly formed hEB. For mass production, only one culture of hESCs/iPSCs for hEB formation was required, and the hEB cell populations could be continuously expanded for more than 20 passages. We were able to scale-up hEB production by about 19 (1∶2 ratio)–369 (1∶6 ratio) folds (mean values) over the conventional method after five weeks of culture (Table S5). We anticipate that the subculturing method of hEBs will therefore be of great benefit for the maintenance of the hEB's regenerative potential and the development of hEB-derived products for applications in therapeutics and drug discovery.

## Supporting Information

Figure S1 **Characterization of hiPSCs derived from human fibroblasts.** (A) Morphology of a representative iPSC colony with high levels of alkaline phosphatase (ALP) and positive immunostaining for two pluripotency markers: OCT4 and Nanog. Cell nuclei were visualized with DAPI. (B) Semi-quantitative RT-PCR analysis of pluripotency gene expression in human foreskin fibroblasts (hFFs), H9 hESs, and hiPSs. GAPDH was used as a loading control. (C) PCR analysis of retroviral integration in genomic DNA from hiPSs. (D) Bisulfite sequencing to measure the DNA methylation status in the promoter region of the OCT4 and NANOG genes in H9 hESs, hiPSs, and hFFs. The promoter regions of OCT4 and NANOG were amplified by PCR using using specific primer sets, and the methylation status was analyzed. Each horizontal row of circles represents an individual sequencing result from one amplicon. Open and black circles indicate demethylated and methylated CpGs, respectively. The proportion of methylated CpGs is indicated.
(TIF)

Figure S2 **Semi-quantitative RT-PCR analysis of the in vitro differentiation assays.** hEBs were formed either with (1) or without enzymatic treatment (collagenase IV) (2) and cultured with (p5, p15, and p17) or without passaging (5 w) for various periods as indicated. (1) 5 w: hEBs formed with enzymatic treatment and cultured for 5 weeks without passaging, (2) 5 w: hEBs formed without enzymatic treatment and cultured for 5 weeks without passaging. (A and B) The spontaneous differentiation potential of H9 hEBs (A) and iPS-hEBs (B) and the directed differentiation potential of H9 hEBs (passage 17) into neural cells, osteoblasts, cardiomyocytes and endothelial cells (C), were determined based on the detection of two pluripotency markers (Oct4 and Nanog) and/or lineage specific markers of the three germ layers using the validated primer sets shown in Table S1.
(TIF)

Figure S3 **Karyotype analysis of long-term-maintained hEBs with passage.** After the H9 hEBs and iPS-hEBs had been serially passaged at a ratio of 1∶4 as indicated, the karyotype was analyzed using the G-banding method.
(TIF)

Table S1 List of primers used in this study.
(DOCX)

Table S2 List of antibodies used in this study.
(DOCX)

Table S3 Expression difference for pluripotency and lineage marker genes in hEB samples comparing to human pluripotenct stem cells.
(DOCX)

Table S4 Cell count data of hEBs cultured under various conditions.
(DOCX)

Table S5 Cell count data of hEBs passaged with different dissociation ratios.
(DOCX)

Text S1 Supplementary Materials and Methods.
(DOC)
