# Supplementary figures and images for: Physical Passaging of Embryoid Bodies Generated from Human Pluripotent Stem Cells
Source: PLoS One. 2011 May 3;6(5):e19134. doi: 10.1371/journal.pone.0019134 (PMC3086884; doi:10.1371/journal.pone.0019134)

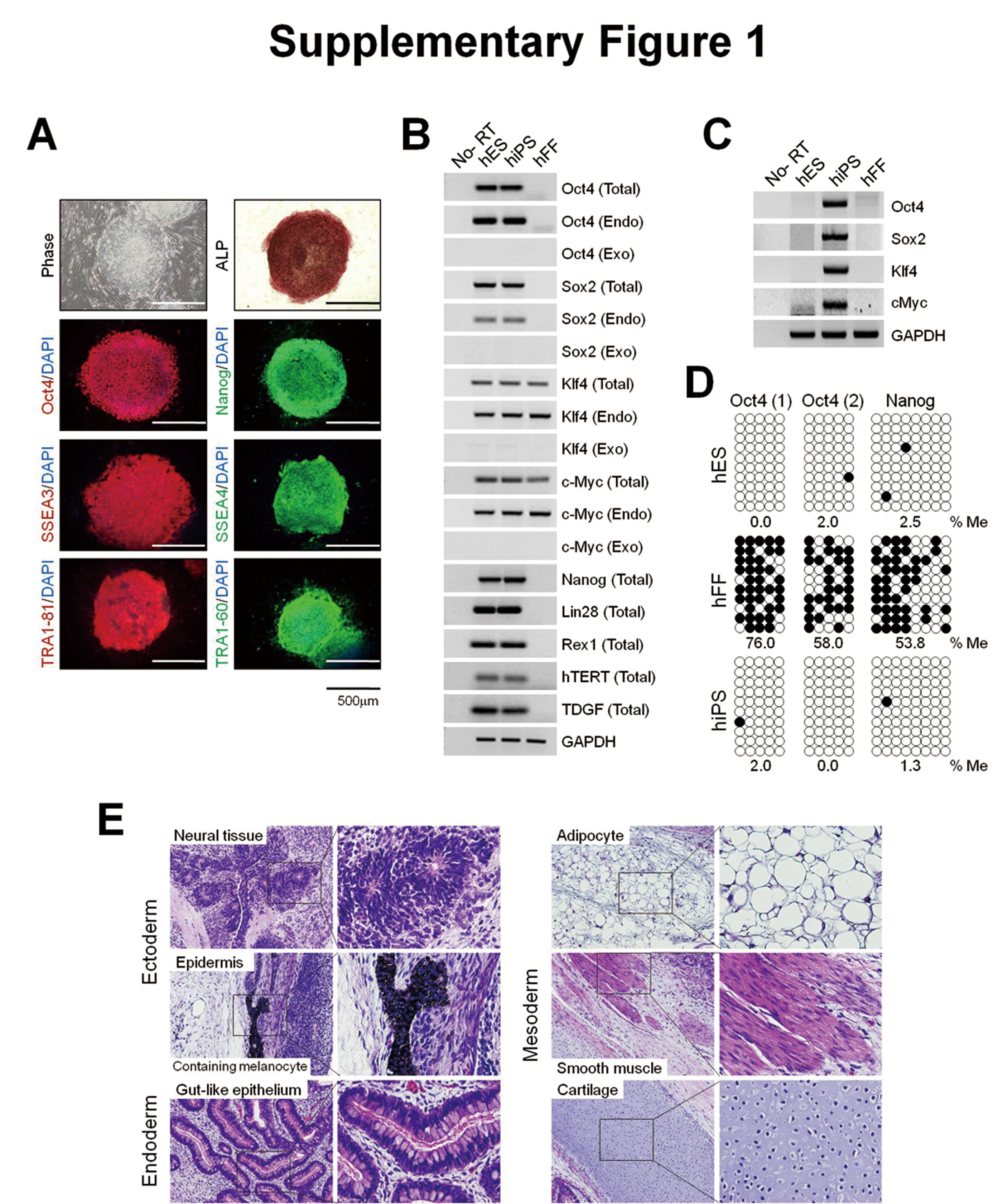

Supplement: Figure S1 — Characterization of hiPSCs derived from human fibroblasts. (A) Morphology of a representative iPSC colony with high levels of alkaline phosphatase (ALP) and positive immunostaining for two pluripotency markers: OCT4 and Nanog. Cell nuclei were visualized with DAPI. (B) Semi-quantitative RT-PCR analysis of pluripotency gene expression in human foreskin fibroblasts (hFFs), H9 hESs, and hiPSs. GAPDH was used as a loading control. (C) PCR analysis of retroviral integration in genomic DNA from hiPSs. (D) Bisulfite sequencing to measure the DNA methylation status in the promoter region of the OCT4 and NANOG genes in H9 hESs, hiPSs, and hFFs. The promoter regions of OCT4 and NANOG were amplified by PCR using using specific primer sets, and the methylation status was analyzed. Each horizontal row of circles represents an individual sequencing result from one amplicon. Open and black circles indicate demethylated and methylated CpGs, respectively. The proportion of methylated CpGs is indicated. (TIF) [file pone.0019134.s001.tif]

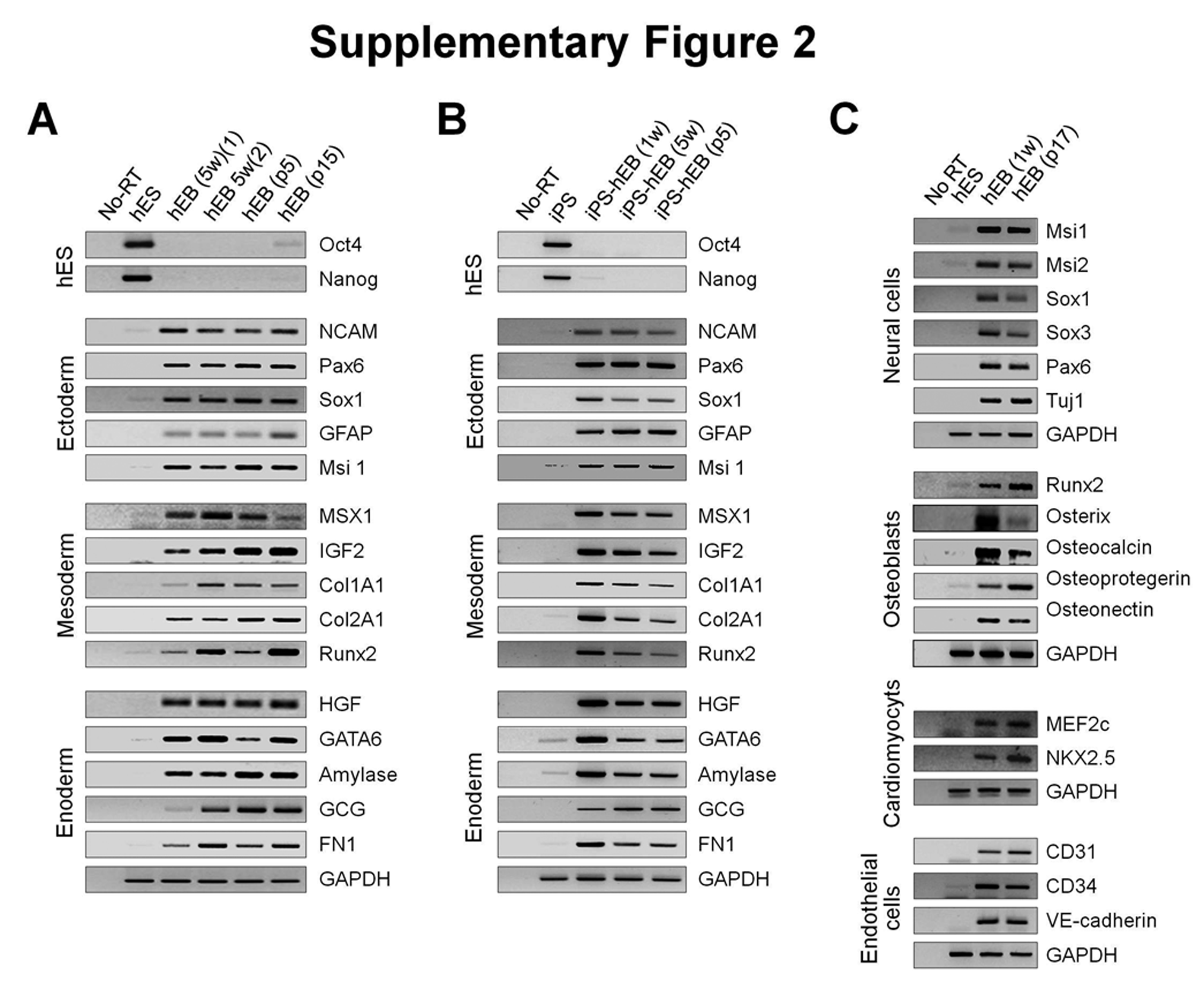

Supplement: Figure S2 — Semi-quantitative RT-PCR analysis of the in vitro differentiation assays. hEBs were formed either with (1) or without enzymatic treatment (collagenase IV) (2) and cultured with (p5, p15, and p17) or without passaging (5 w) for various periods as indicated. (1) 5 w: hEBs formed with enzymatic treatment and cultured for 5 weeks without passaging, (2) 5 w: hEBs formed without enzymatic treatment and cultured for 5 weeks without passaging. (A and B) The spontaneous differentiation potential of H9 hEBs (A) and iPS-hEBs (B) and the directed differentiation potential of H9 hEBs (passage 17) into neural cells, osteoblasts, cardiomyocytes and endothelial cells (C), were determined based on the detection of two pluripotency markers (Oct4 and Nanog) and/or lineage specific markers of the three germ layers using the validated primer sets shown in Table S1. (TIF) [file pone.0019134.s002.tif]

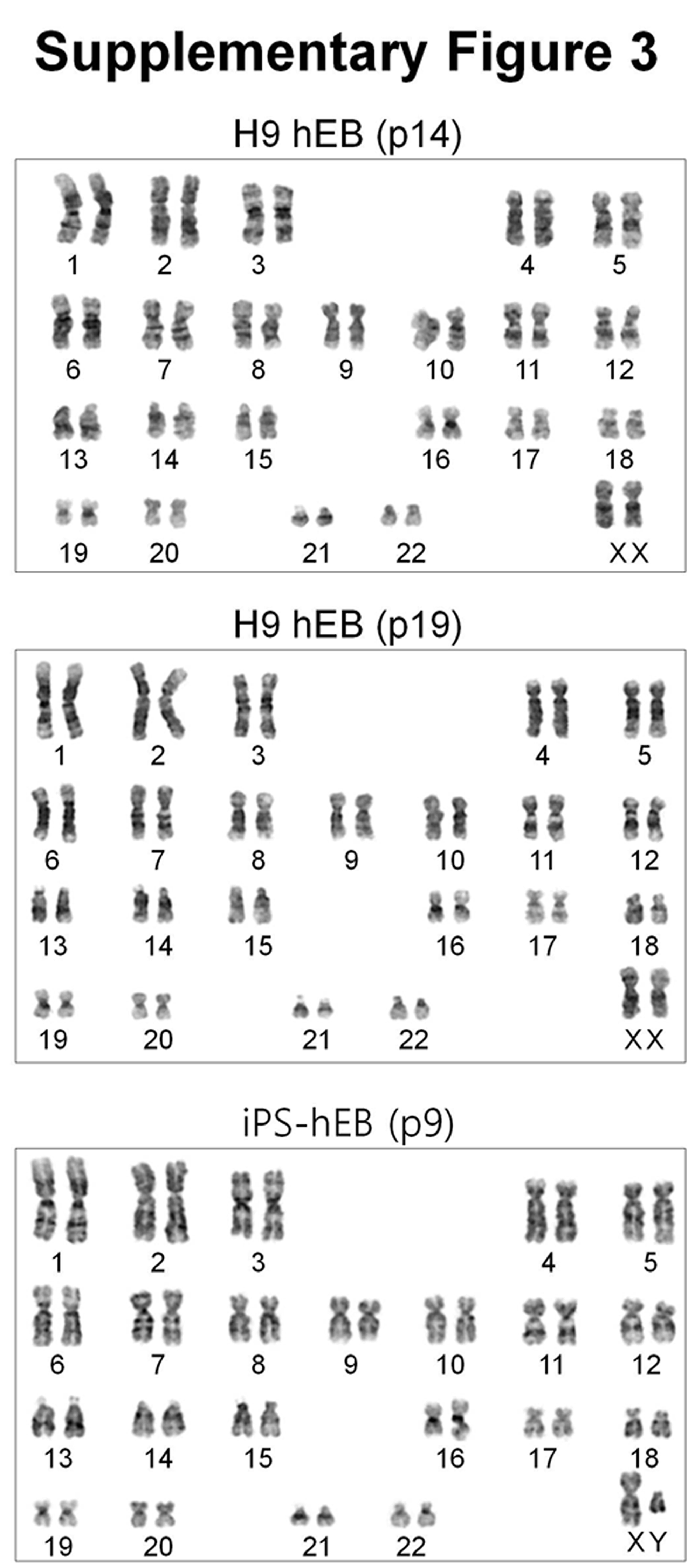

Supplement: Figure S3 — Karyotype analysis of long-term-maintained hEBs with passage. After the H9 hEBs and iPS-hEBs had been serially passaged at a ratio of 1∶4 as indicated, the karyotype was analyzed using the G-banding method. (TIF) [file pone.0019134.s003.tif]
