# Supplementary material for: Physical Passaging of Embryoid Bodies Generated from Human Pluripotent Stem Cells
Source: PLoS One. 2011 May 3;6(5):e19134. doi: 10.1371/journal.pone.0019134 (PMC3086884; doi:10.1371/journal.pone.0019134)
Supplement: Table S1 — List of primers used in this study. (DOCX) [file pone.0019134.s004.docx]

**Table S1**. List of primers used in this study

| Gene | Primer (Forward) | Primer (Reverse) | Accession No. |
| --- | --- | --- | --- |
| *Total OCT4* | GAGAAGGATGTGGTCCGAGTGTG | CAGAGGAAAGGACACTGGTCCC | NM_002701 |
| *Total SOX2* | AGAACCCCAAGATGCACAAC | ATGTAGGTCTGCGAGCTGGT | NM_003106 |
| *Total KLF4* | ACCCTGGGTCTTGAGGAAGT | ACGATCGTCTTCCCCTCTTT | NM­_004235 |
| *Total c-Myc* | CCTACCCTCTCAACGACAGC | CTCTGACCTTTTGCCAGGAG | NM_002467 |
| *Endo OCT4* | GACAGGGGGAGGGGAGGAGCTAGG | CTTCCCTCCAACCAGTTGCCCCAAAC |  |
| *Endo SOX2* | GGGAAATGGGAGGGGTGCAAAAGAGG | TTGCGTGAGTGTGGATGGGATTGGTG |  |
| *Endo KLF4* | ACGATCGTGGCCCCGGAAAAGGACC | TGATTGTAGTGCTTTCTGGCTGGGCTCC |  |
| *Endo c-Myc* | GCGTCCTGGGAAGGGAGATCCGGAGC | TTGAGGGGCATCGTCGCGGGAGGCTG |  |
| For transgene and genomic integration | | | |
| *Trans OCT4* | GAGAAGGATGTGGTCCGAGTGTG | CCCTTTTTCTGGAGACTAAATAAA |  |
| *Trans SOX2* | GGCACCCCTGGCATGGCTCTTGGCTC | TTATCGTCGACCACTGTGCTGCTG |  |
| *Trans KLF4* | ACGATCGTGGCCCCGGAAAAGGACC | TTATCGTCGACCACTGTGCTGCTG |  |
| *Trans c-Myc* | CAACAACCGAAAATGCACCAGCCCCAG | TTATCGTCGACCACTGTGCTGCTG |  |
| hESC markers | | | |
| *Nanog* | CAAAGGCAAACAACCCACTT | ATTGTTCCAGGTCTGGTTGC | NM_024865 |
| *Lin28* | GAAGCGCAGATCAAAAGGAG | GGGTAGGGCTGTGGATTTCT | NM_024674 |
| *hTERT* | CGGAAGAGTGTCTGGAGCAA | GGATGAAGCGGAGTCTGGA | NM_198255 |
| *TDGF* | TCCTTCTACGGACGGAACTG | AGAAATGCCTGAGGAAAGCA | NM_003212 |
| *Rex1* | AATGCGTCATAAGGGGTGAG | TCAATGCCAGGTATTCCTCC | NM_174900 |
| Ectoderm lineage markers | | | |
| *NCAM* | AGGAGACAGAAACGAAGCCA | GGTGTTGGAAATGCTCTGGT | NM_000615 |
| *Pax6* | GCCAGCAACACACCTAGTCA | TGTGAGGGCTGTGTCTGTTC | NM_000280 |
| *GFAP* | CCTCTCCCTGGCTCGAATG | GGAAGCGAACCTTCTCGATGTA | NM_002055 |
| *Sox1* | GGGAAAACGGGCAAAATAAT | CCATCTGGGCTTCAAGTGTT | NM_005986 |
| *Sox3* | GACGCCTTGTTTAGCTTTGC | TTCTCCCATTCACTCCTTGG | NM_005634 |
| *MSI1* | ACCCCCACATTCTCTCACTG | AAACCCAAAACACGAACAGC | NM_002442 |
| *MSI2* | TTTGTAGGCGGGTTATCTGC | GCCATAGCTTGGAGCAAATC | NM_138962 |
| *Tuj1* | ACCTCAACCACCTGGTATCG | GGGTACCACTCCACGAAGTA | NM_006086 |
| Mesoderm lineage markers | | | |
| *MSX1* | TCCTCAAGCTGCCAGAAGAT | TACTGCTTCTGGCGGAACTT | NM_002448 |
| *IGF2* | CAGACCCCCAAATTATCGTG | GCCAAGAAGGTGAGAAGCAC | NM_000612 |
| *Col1A1* | GGACACAATGGATTGCAAGG | TAACCACTGCTCCACTCTGG | NM_000088 |
| *Col2A1* | CCGCGGRGAGCCATGATTCG | CAGGCCCAGGAGGTCCTTTGGG | NM_001844 |
| *Runx2* | CGGCAAAATGAGCGACGTG | CACCGAGCACAGGAAGTTG | NM_004348 |
| *Osterix* | CCCAGGCAACACTCCTACTC | GGCTGGATTAAGGGGAGCAAA | NM_152860 |
| *Osteocalcin* | CACTCCTCGCCCTATTGGC | GCCTGGGTCTCTTCACTACCT | NM_199173 |
| *Osteoprotegerin* | AGCACCCTGTAGAAAACACAC | ACACTAAGCCAGTTAGGCGTAA | NM_002546 |
| *Osteonectin* | AGCACCCCATTGACGGGTA | GGTCACAGGTCTCGAAAAAGC | NM_003118 |
| *MEF2c* | AGATACCCACAACACACCACGCGCC | ATCCTTCAGAGAGTCGCATGC | NM_002397 |
| *NKX2.5* | CATTTACCCGGGAGCCTACG | GCTTTCCGTCGCCGCCGTGCGCGTG | NM_004387 |
| Endoderm lineage markers | | | |
| *HGF* | gcatcaaatgtcagccctgg | caacgctgacatggaattcc | NM_000601 |
| *GATA6* | CCATGACTCCAACTTCCACC | ACGGAGGACGTGACTTCGGC | NM_005257 |
| *Amylase* | GCTGGGCTCAGTATTCCCCAAAT | GACGACAATCTCTGACCTGAGTAG | NM_000699 |
| *GCG* | acgaccattcccaacacac | catcaaccactgcacaaaatc | NM_002054 |
| *FN1* | ccacttccccttcctatacaac | acgaccattcccaacacac | NM_212482 |
| *CD31* | atcatttctagcgcatggcctggt | atttgtggagggcgaggtcataga | NM_000442 |
| *CD34* | aaatcctcttcctctgaggctgga | aagaggcagctggtgataagggtt | NM_001025109 |
| *VE-cadherin* | tggagaagtggcatcagtcaacag | tctacaatcccttgcagtgtgag | NM_001795 |
| Housekeeping gene | | | |
| *GAPDH* | GAAGGTGAAGGTCGGAGTC | GAAGATGGTGATGGGATTTC | NM_002046 |
