# Supplementary material for: Physical Passaging of Embryoid Bodies Generated from Human Pluripotent Stem Cells
Source: PLoS One. 2011 May 3;6(5):e19134. doi: 10.1371/journal.pone.0019134 (PMC3086884; doi:10.1371/journal.pone.0019134)
Supplement: Table S2 — List of antibodies used in this study. (DOCX) [file pone.0019134.s005.docx]

**Table S2**. List of antibodies used in this study

| Antibodies | Catalog No. | Company | Dilution |
| --- | --- | --- | --- |
| ***In vitro* differentiation** |  |  |  |
| anti-TUJ1 | PRB-435P | Covance | 1:500 for immunostaining |
| anti-NESTIN | MAB5326 | Chemicon | 1:100 for immunostaining |
| anti-α-FP | A8453 | Sigma | 1:500 for immunostaining |
| anti-SOX17 | MAB1924 | R&D | 1:50 for immunostaining |
| anti-α-SMA | A5228 | Sigma | 1:400 for immunostaining |
| anti-desmin | AB907 | Chemicon | 1:30 for immunostaining |
| **Directed differentiation** |  |  |  |
| anti-Osteocalcin | MAB1419 | R&D | 1:100 for immunostaining |
| anti-Cardiac Troponin T | MAB1874 | R&D | 1:10 for immunostaining |
| anti-MHC | 05-833 | Upstate | 1:100 for immunostaining |
| anti-MAP2 | AB5622 | Chemicon | 1:500 for immunostaining |
| anti-GFAP | MAB3402 | Chemicon | 1:200 for immunostaining |
| anti- O4 | MAB345 | Chemicon | 1:50 for immunostaining |
| anti-PECAM-1 | 3528 | Cell signaling | 1:100 for immunostaining |
| anti- VE cadherin | Ab7047 | Abcam | 1:100 for immunostaining |
| ***ESC markers*** |  |  |  |
| anti-Oct4 | sc-9081 | Santa Cruz Biotechnology | 1:50 for immunostaining |
| anti-Nanog | sc-33759 | Santa Cruz Biotechnology | 1:200 for immunostaining |
