# Supplementary material for: Physical Passaging of Embryoid Bodies Generated from Human Pluripotent Stem Cells
Source: PLoS One. 2011 May 3;6(5):e19134. doi: 10.1371/journal.pone.0019134 (PMC3086884; doi:10.1371/journal.pone.0019134)
Supplement: Table S3 — Expression difference for pluripotency and lineage marker genes in hEB samples comparing to human pluripotenct stem cells. (DOCX) [file pone.0019134.s006.docx]

| Table S3. Expression difference for pluripotency and lineage marker genes in hEB samples comparing to human pluripotenct stem cells. | | | | | | | | | | | | |
| --- | --- | --- | --- | --- | --- | --- | --- | --- | --- | --- | --- | --- |
| Gene Symbol | Description | Genbank | Relative Fold Change (Log2) | | | | | | | | | |
|  |  |  | *vs.* hESC | | | | | |  | *vs.* hiPSC | | |
|  |  |  | 1w | 2w | 5w | p1 | p5 | p15 |  | 1w | 5w | p5 |
| *Stem cell markers* | | | | | | | | | | | | |
| NANOG | Nanog homeobox | NM_024865 | -4.3 | -4.2 | -6.1 | -3.5 | -6.6 | -5.7 |  | -2.4 | -6.8 | -9.0 |
| POU5F1 | POU domain, class 5, transcription factor 1 | NM_002701 | -4.0 | -3.5 | -6.6 | -4.1 | -6.6 | -6.1 |  | -2.4 | -7.5 | -6.5 |
| LEFTY1 | left-right determination factor 1 | NM_020997 | -6.4 | -6.6 | -4.2 | -6.6 | -5.3 | -5.0 |  | -5.5 | -7.1 | -5.6 |
| LEFTY2 | left-right determination factor 2 | NM_003240 | -5.0 | -3.3 | -3.9 | -4.5 | -2.7 | -3.7 |  | -2.2 | -2.2 | -2.2 |
| TDGF1 | teratocarcinoma-derived growth factor 1 | NM_003212 | -2.8 | -5.2 | -5.9 | -6.5 | -3.3 | -5.8 |  | -0.9 | -4.5 | -5.7 |
| GAL | galanin | NM_015973 | -3.0 | -2.3 | -3.2 | -2.9 | -3.5 | -5.6 |  | -1.5 | -4.5 | -3.3 |
| FGFR1 | fibroblast growth factor receptor 1 (fms-related tyrosine kinase 2, Pfeiffer syndrome) | NM_023111 | -2.8 | -3.5 | -3.5 | 0.0 | -3.3 | -4.5 |  | -0.8 | -2.6 | -2.9 |
| ZFP42 | zinc finger protein 42 homolog (mouse) | NM_174900 | -2.4 | -1.5 | -6.0 | -2.3 | -6.6 | -6.5 |  | -0.5 | -5.3 | -10.1 |
| GDF3 | growth differentiation factor 3 | NM_020634 | -1.3 | -4.4 | -3.3 | -5.3 | -4.6 | -4.9 |  | -2.9 | -6.1 | -5.9 |
| SALL4 | sal-like 4 (Drosophila) | NM_020436 | -0.8 | -0.7 | -5.9 | -0.6 | -4.5 | -4.7 |  | 0.5 | -5.1 | -3.6 |
| SOX2 | SRY (sex determining region Y)-box 2 | NM_003106 | -0.8 | -0.5 | -1.9 | -0.5 | -0.5 | -1.1 |  | 0.3 | -0.7 | -0.4 |
| LIN28 | lin-28 homolog (C. elegans) | NM_024674 | 0.1 | 0.1 | -3.7 | 0.2 | -2.9 | -3.3 |  | 0.1 | -1.9 | -2.7 |
| DNMT3B | DNA (cytosine-5-)-methyltransferase 3 beta | NM_175850 | 0.1 | 0.1 | -1.3 | -1.1 | -2.4 | -3.1 |  | -0.2 | -2.6 | -1.3 |
| *Ectoderm markers* | | | | | | | | | | | | |
| NEUROD1 | neurogenic differentiation 1 | NM_002500 | 3.8 | 3.7 | 5.6 | 2.5 | 6.0 | 1.4 |  | 0.0 | 1.7 | 2.5 |
| DDC | dopa decarboxylase (aromatic L-amino acid decarboxylase) | NM_000790 | 3.5 | 2.0 | 4.3 | 3.9 | 4.5 | 1.6 |  | 2.4 | 2.2 | 3.1 |
| PAX6 | paired box gene 6 (aniridia, keratitis) | NM_000280 | 2.5 | 3.4 | 3.9 | 2.7 | 3.2 | 2.4 |  | 3.1 | 2.2 | 4.0 |
| GAD2 | glutamate decarboxylase 2 (pancreatic islets and brain, 65kDa) | NM_000818 | 3.1 | 2.7 | 3.6 | 3.8 | 5.5 | 4.0 |  | -1.4 | 1.7 | 2.8 |
| MAPT | microtubule-associated protein tau | NM_016835 | 1.6 | 5.0 | 4.6 | 3.8 | 7.3 | 8.6 |  | -2.1 | 5.6 | 5.8 |
| TH | tyrosine hydroxylase | NM_199292 | 1.1 | 1.6 | 2.5 | 5.2 | 2.5 | 2.0 |  | 1.2 | 2.2 | 2.0 |
| CXCR4 | chemokine (C-X-C motif) receptor 4 | NM_001008540 | 1.8 | 2.3 | 2.5 | 2.2 | 2.3 | 0.7 |  | 1.0 | 2.5 | 2.6 |
| SLC6A4 | solute carrier family 6 (neurotransmitter transporter, serotonin), member 4 | NM_001045 | 1.1 | 0.7 | -0.2 | 0.1 | 1.4 | 0.9 |  | 0.7 | 1.1 | 0.1 |
| INA | internexin neuronal intermediate filament protein, alpha | NM_032727 | 1.3 | 0.0 | 3.0 | 1.5 | 3.2 | 3.4 |  | 0.1 | 3.2 | 3.4 |
| SLC32A1 | solute carrier family 32 (GABA vesicular transporter), member 1 | NM_080552 | 0.6 | 3.5 | 3.7 | 4.5 | 5.5 | 1.3 |  | 0.8 | 5.0 | 7.2 |
| NCAM1 | Neural cell adhesion molecule 1 | NM_001076682 | 0.1 | 1.7 | 1.1 | 0.7 | 1.5 | 1.3 |  | 1.1 | 1.6 | 1.0 |
| TUBB3 | tubulin, beta 3 | NM_006086 | -0.4 | 1.1 | 0.9 | 1.6 | 1.7 | 2.3 |  | 0.2 | 1.9 | 2.5 |
| VIM | vimentin | NM_003380 | -0.3 | 0.6 | -0.2 | 0.4 | 0.5 | -0.4 |  | 0.8 | 0.3 | 0.9 |
| SOX1 | SRY (sex determining region Y)-box 1 | NM_005986 | 0.6 | 0.6 | 2.2 | 1.3 | 0.6 | 1.0 |  | -0.9 | 3.1 | 3.7 |
| MAP1A | microtubule-associated protein 1A | NM_002373 | 0.0 | 0.5 | 0.3 | 1.4 | 2.4 | 2.0 |  | -0.9 | 0.8 | 1.7 |
| *Endoderm markers* | | | | | | | | | | | | |
| GATA6 | GATA binding protein 6 | NM_005257 | 5.0 | 2.7 | 3.2 | 3.6 | -0.1 | -0.7 |  | 0.4 | 1.3 | -3.0 |
| FOXA2 | forkhead box A2 | NM_021784 | 3.5 | 0.8 | 3.7 | 2.4 | 2.4 | 2.2 |  | 1.9 | -0.3 | 0.5 |
| GAD2 | glutamate decarboxylase 2 (pancreatic islets and brain, 65kDa) | NM_000818 | 3.1 | 2.7 | 3.6 | 3.8 | 5.5 | 4.0 |  | -1.4 | 1.7 | 2.8 |
| LAMA1 | laminin, alpha 1 | NM_005559 | 2.8 | 2.6 | 0.5 | 0.0 | 1.1 | 0.7 |  | 2.2 | 0.5 | 2.3 |
| SERPINA1 | serpin peptidase inhibitor, clade A (alpha-1 antiproteinase, antitrypsin), member 1 | NM_001002236 | 1.7 | 7.0 | 6.8 | 4.1 | 1.6 | 1.6 |  | 5.8 | 5.6 | 2.2 |
| SMAD2 | SMAD family member 2 | NM_001003652 | 1.6 | 1.1 | 0.7 | 0.1 | 0.7 | 0.8 |  | 1.2 | 0.3 | 0.3 |
| HGF | hepatocyte growth factor (hepapoietin A; scatter factor) | ENST00000222390 | 0.7 | 1.8 | 0.7 | 0.7 | 2.2 | 0.2 |  | -0.1 | 0.0 | 2.4 |
| LAMB1 | laminin, beta 1 | NM_002291 | 0.7 | 1.2 | 0.7 | 0.9 | -0.2 | -0.4 |  | 0.1 | 0.5 | -2.2 |
| AFP | alpha-fetoprotein | NM_001134 | 0.5 | 2.7 | 1.2 | 1.5 | 0.0 | 1.0 |  | 5.1 | 4.2 | 0.4 |
| HES1 | hairy and enhancer of split 1, (Drosophila) | NM_005524 | 0.4 | 2.2 | 1.9 | 1.5 | 1.7 | 1.0 |  | 1.4 | 1.3 | 1.0 |
| GCG | glucagon | NM_002054 | 0.2 | 1.9 | 1.2 | 0.8 | 1.6 | 1.4 |  | 0.3 | 0.0 | 0.1 |
| HHEX | hematopoietically expressed homeobox | NM_002729 | 0.2 | 1.9 | 1.2 | 0.8 | 1.7 | 1.5 |  | 1.3 | -2.4 | -2.3 |
| HNF4A | hepatocyte nuclear factor 4, alpha | NM_001030004 | 0.2 | 1.9 | 1.3 | 0.8 | 1.7 | 1.5 |  | 0.9 | 0.3 | 0.8 |
| DCN | decorin | NM_001920 | -1.3 | 1.1 | 0.5 | 0.4 | 0.5 | 1.8 |  | 2.8 | 8.6 | 3.5 |
| INS | insulin | NM_000207 |  |  |  |  |  |  |  | -0.6 | 1.0 | 1.2 |
| *Mesoderm markers* | | | | | | | | | | | | |
| IGF2 | insulin-like growth factor 2 (somatomedin A) | NM_000612 | 3.4 | 3.6 | 3.6 | 3.2 | 2.5 | -0.5 |  | 5.8 | 6.5 | 4.2 |
| MESP1 | mesoderm posterior 1 homolog (mouse) | NM_018670 | 2.9 | 0.9 | 3.0 | 2.1 | 2.2 | 2.6 |  | 0.1 | 2.2 | 0.5 |
| HAND1 | heart and neural crest derivatives expressed 1 | NM_004821 | 2.5 | 2.2 | 0.8 | 2.7 | 0.3 | 0.6 |  | 3.4 | 2.5 | -1.1 |
| MYF6 | myogenic factor 6 (herculin) | NM_002469 | 2.3 | 2.1 | 1.6 | 1.4 | 1.1 | 0.7 |  | 3.3 | 3.5 | 2.2 |
| T | T, brachyury homolog (mouse) | NM_003181 | 2.1 | -0.7 | 2.7 | 0.2 | 2.9 | 2.7 |  | 2.8 | 0.5 | 1.5 |
| HOXD13 | homeobox D13 | NM_000523 | 0.6 | 1.9 | 2.1 | 2.0 | 1.6 | 1.4 |  | 0.2 | -0.1 | 0.0 |
| SHH | sonic hedgehog homolog (Drosophila) | NM_000193 | 0.9 | 0.2 | 2.6 | 1.2 | 0.6 | 1.7 |  | 0.5 | 1.4 | 0.3 |
| TNNT2 | troponin T type 2 (cardiac) | NM_000364 | 0.7 | -0.6 | 1.8 | 0.2 | 0.6 | 0.2 |  | 0.8 | 1.3 | 0.4 |
| NPPA | natriuretic peptide precursor A | NM_006172 | 0.2 | 5.5 | 5.4 | 6.3 | 6.3 | 6.2 |  | 0.2 | 5.6 | 5.9 |
| SLC2A2 | solute carrier family 2 (facilitated glucose transporter), member 2 | NM_000340 | 0.2 | 2.0 | 2.1 | 0.8 | 1.7 | 1.5 |  | 0.0 | 0.0 | 1.1 |
| FABP4 | fatty acid binding protein 4, adipocyte | NM_001442 | 0.2 | 1.8 | 1.2 | 0.7 | 1.6 | 1.4 |  | 0.7 | 0.0 | 0.1 |
| GSC | goosecoid | NM_173849 | -0.6 | 1.2 | 0.5 | 0.1 | 0.9 | 0.8 |  | -0.9 | 1.4 | -0.5 |
