# Supplementary material for: Physical Passaging of Embryoid Bodies Generated from Human Pluripotent Stem Cells
Source: PLoS One. 2011 May 3;6(5):e19134. doi: 10.1371/journal.pone.0019134 (PMC3086884; doi:10.1371/journal.pone.0019134)
Supplement: Table S4 — Cell count data of hEBs cultured under various conditions. (DOCX) [file pone.0019134.s007.docx]

Table S4. Cell count data of hEBs cultured under various conditions. Cell counts are mean values ±SD of three independent experiments. Fold increases were displayed by mean values.

| Culture conditions | | | Cell counts per a single hES clump at day 0 | Cell counts  per a single hEB  at day 14 | Numbers of hEB aggregates at day 14 | Total cell counts derived from a single hES clump | Fold increases in cell counts (hEB at day 14  *vs* hES clump at day 0 |
| --- | --- | --- | --- | --- | --- | --- | --- |
|  | hEB clumps with uniform size  (Step 1) | Passages  (Step 2) |  |  |  |  |  |
| Fig.1A*i* | No | No | 1493±653 | (*a*) 8376±874 | (*a’*)1 | (*a* x *a’*)  8376 | 5.6 |
| Fig.1A*ii* | Yes | No | 1687±187 | (*b*)10330±341 | (*b’*)1 | (*b* x *b’*)  10330 | 6.1 |
| Fig.1A*iii* | Yes | Yes  (1:4 ratio) |  | (*c*)7124±342 | (*c’*)4 | (*c* x *c’*)  28496 | 16.8 |
