# Supplementary material for: Physical Passaging of Embryoid Bodies Generated from Human Pluripotent Stem Cells
Source: PLoS One. 2011 May 3;6(5):e19134. doi: 10.1371/journal.pone.0019134 (PMC3086884; doi:10.1371/journal.pone.0019134)
Supplement: Table S5 — Cell count data of hEBs passaged with different dissociation ratios. (DOCX) [file pone.0019134.s008.docx]

Table S5. Cell count data of hEBs passaged with different dissociation ratios. Cell counts are mean values ±SD of three independent experiments. Fold increases were displayed by mean values (minimum/maximum values).

| Culture conditions | Cell counts  per a single hEB  at day 7 | Dissociation  ratios | Incubation days between passages | Cell counts  per a single hEB  at passage 5 | Numbers  of hEB aggregates at passage 5 | Total cell counts derived from a single hES clump at passage 5 | Fold increases in cell counts ( passaging method  *vs c*onventional method) |
| --- | --- | --- | --- | --- | --- | --- | --- |
| Passaging method  (Fig.1A*iii*) | 7,587±0.61 | 1:2 | 6 | (*a*) 7,944±257 | (*a’*)  18±6 | (*a* x *a’*)  142,992 | 18.9  (12.6/25.3) |
|  |  | 1:4 | 8 | (*b*)  7,444±342 | (*b’*)  122±57 | (*b* x *b’*)  908,168 | 120.3  (64.1/176.5) |
|  |  | 1:6 | 14 | (*c*)  7,166±254 | (*c’*)  389±91 | (*c* x *c’*)  2,787,574 | 369.3  (282.9/455.7) |
| Conventional method (Fig.1A*ii*) |  | - | - | 7548±715 | 1 | 7548 | - |
