# Supplementary material for: Physical Passaging of Embryoid Bodies Generated from Human Pluripotent Stem Cells
Source: PLoS One. 2011 May 3;6(5):e19134. doi: 10.1371/journal.pone.0019134 (PMC3086884; doi:10.1371/journal.pone.0019134)
Supplement: Text S1 — Supplementary Materials and Methods. (DOC) [file pone.0019134.s009.doc]

**Supplementary Materials and Methods**

*hiPSC generation*

Human newborn foreskin fibroblasts (hFFs, catalog number CRL-2097; ATCC, Manassas, VA) were maintained in Dulbecco's Modified Eagle Medium (DMEM) containing 10% fetal bovine serum (FBS, Invitrogen, Carlsbad, CA), 1% non-essential amino acids (NEAA, Invitrogen), 1 mM L-glutamine (Invitrogen) and 0.1 mM β-mercaptoethanol (Sigma). For iPSC generation, human foreskin fibroblasts (hFFs; 1 × 10^5^ cells per well) were transduced with pMX-based retroviruses encoding human Oct4, Sox2, Klf4, and c-Myc (Addgene Inc., Cambridge, MA). Five days after transduction, the hFFs were replated in gelatin-coated 6-well dishes (5-6 × 10^4^ cells per well) that had been pre-plated with MEF feeders. The following day, the medium was replaced with hESC medium supplemented with 10 ng/ml bFGF. The medium was changed every other day. Colonies that exhibited a hESC-like morphology were picked 20-23 days after transduction and transferred into 12-well dishes that had been pre-plated with MEF feeders. Selected hiPSC colonies were expanded under standard hESC culture conditions and used for further analyses.

*PCR analysis*

Semi-quantitative RT-PCR was performed using the Platinum Taq SuperMix kit (Invitrogen) under the following conditions: 3 min at 94°C, followed by 30 cycles of 30 s at 94°C, 30 s at 60°C, and 30 s at 72°C, and a final extension for 10 min 72°C. Glyceraldehyde-3-phosphate dehydrogenase (GAPDH) was used as the endogenous control. Quantitative real-time PCR was also performed using Power SYBR Green PCR Master Mix (Applied Biosystems) and the ABI 7500 Real-Time PCR System (Applied Biosystems). After activation of the Taq polymerase at 95°C for 15 min, the reactions were denatured at 95°C for 15 sec and then annealed and elongated at 60°C for 1 min; this process was repeated for 50 cycles. The PCR products were separated in a 1.5% agarose gel containing ethidium bromide and visualized with a Gel Doc EQ system (Bio-Rad, Watford, UK). All experiments were run in triplicate, and the CT value for each target gene was determined using the software provided by the manufacturer and normalized to the expression level of GAPDH. The primer sequences used in the present study are shown in Supplementary Table 1.

*Western blot analysis*

hEBs were lysed in RIPA buffer containing 50 mM Tris, pH 8.0, 150 mM NaCl, 1% NP-40, 0.1% SDS, 0.5% deoxycholic acid, 1 mM PMSF, and a cocktail of protease inhibitors (Roche Applied Science, Indianapolis, IN) for 15 min on ice and centrifuged at 20,000 × *g* for 10 min at 4°C. The supernatant was re-centrifuged for 10 min, and the protein concentrations were determined using the BCA protein assay kit (Pierce, Rockford, IL). Proteins (20 μg) were separated by SDS polyacrylamide gel electrophoresis (PAGE) and electrotransferred to polyvinylidene fluoride (PVDF) membranes (Millipore Corp, Bedford, MA). The membranes were blocked in PBS containing 0.1% Tween-20 and 5% non-fat milk for 2 hrs at RT and then probed with specific primary antibodies for 1 hr. After washing, the membranes were incubated with HRP-conjugated secondary antibodies (Amersham, Arlington Heights, IL). The bands were visualized using the ECL Advance Kit (Amersham) and LAS**-**3000 **(**Fujifilm, Tokyo, Japan). The density of the bands was analyzed using Image Gauge software (Fuji Photo Film GMBH, Düsseldorf) and normalized to those of the loading control (β-actin).

*BrdU incorporation assays*

hEBs were trypsinized and grown on Matrigel-coated 4-well Lab-Tek chamber slides for 4 days for the 5-bromo-20-deoxyuridine (BrdU; BD Pharmingen, San Diego, CA) incorporation assays. For BrdU incorporation, the cells were incubated in the presence of 10 μM BrdU for 1 hr at 37°C. After washing with PBS, the cells were fixed with 4% paraformaldehyde for 15 min and then incubated in 1 N HCl for 15 min at RT. The samples were then washed and incubated with 0.1 M sodium tetraborate for 15 min. After washing, the cells were incubated with anti-BrdU antibody (1:100; BD Pharmingen) for 60 min in PBS/3% BSA and then with FITC-conjugated secondary antibody (Invitrogen) for 30 min. Nuclei were visualized with DAPI. After washing in PBS, the coverslips were mounted and examined under an Olympus fluorescence microscope (IX51, Olympus, Japan). The mean ± SE number of BrdU-positive cells per field of vision was determined. At least 4 fields of vision per coverslip were counted.

*Cell-cycle analysis*

hEBs were dissociated into single cells with 0.1% trypsin/EDTA solution, and viable cell numbers were counted using the trypan blue exclusion method. Approximately 1 × 10^6^ cells were used for the analysis. The cells were washed in PBS, fixed in ice-cold 100% ethanol and incubated for 30 min at 4°C. The cells were then washed in PBS, incubated in 1 ml staining solution (20 μg/ml propidium iodide/10 μg/ml RNaseA) for 30 min at room temperature and examined using a flow cytometry (BD Biosciences, Franklin Lakes, NJ).

*Apoptosis assays*

Apoptosis assays for the hEBs were analyzed by flow cytometry using the Annexin-V-FITC Apoptosis Detection Kit I (BD Biosciences) according to the manufacturer’s instructions. Briefly, single-cell dissociated hEBs were resuspended in binding buffer at 1 × 10^6^ cells/ml and stained with Annexin-V and propidium iodide for 15 min. The cells were then diluted 1:1 in binding buffer, filtered, and counted immediately using a flow cytometer (BD Biosciences).

*Protein oxidation analysis*

Carbonyl groups resulting from protein oxidation were detected using the OxyBLOT™ Protein Oxidation Detection Kit (Millipore, Billerica, MA) according to the manufacturer's instructions. Total proteins were isolated from prepared hEBs using RIPA buffer containing 50 mM Tris, pH 8.0, 150 mM NaCl, 1% NP-40, 0.1% SDS, 0.5% deoxycholic acid, 1 mM PMSF, and a cocktail of protease inhibitors (Roche Applied Science, Indianapolis, IN). The protein concentration was determined using the BCA method (Pierce Biotechnology, Rockford, IL). After derivatization of the carbonyl groups in the protein side chains by treatment with 2,4-dinitrophenylhydrazine, the proteins were separated in 10% SDS-PAGE gels and transferred to a polyvinylidene difluoride (PVDF) membrane (Immobilon-P, Millipore). The membrane was then treated with anti-dinitrophenyl primary antibody (1:150) followed by goat anti-rabbit IgG (1:300). Immunoreactive proteins were visualized using the ECL Advance Kit (Amersham) and LAS**-**3000 (Fujifilm, Tokyo, Japan).

*Microarray analysis*

Total RNA from hESCs, hiPSCs, and hEBs was extracted using the RNeasy Mini Kit (Qiagen), labeled with Cy3 and hybridized to Agilent Whole Human Genome 4X44K Microarrays (one-color platform) according to the manufacturers’ protocols. The hybridized images were scanned using the Agilent DNA microarray scanner and quantified with Feature Extraction software (Agilent Technology, Palo Alto, CA). Normalization of the data and determination of the fold-change in genes were performed using GeneSpring GX 7.3 (Agilent Technology, USA). The averages of the normalized ratios were calculated by dividing the average of the normalized signal channel intensity by the average of the normalized control channel intensity.

*Karyotype analysis*

Long-term-maintained hEBs were processed for chromosomal G-band analysis by GenDix, Inc. (Seoul, Korea). A representative image captured by ChIPS-Karyo (Chromosome Image Processing System, GenDix, Inc.) is shown.

*Alkaline phosphatase staining*

Alkaline phosphatase (ALP) staining was performed using a commercially available ALP kit according to the manufacturer’s instructions (Sigma). Images of ALP-positive cells were recorded using an HP Scanjet G4010. Bright field images were obtained using an Olympus microscope (IX51, Olympus, Japan).

*PCR analysis of genomic integration*

Genomic DNA samples were isolated using the DNeasy Kit (Qiagen, Valencia, CA). Each PCR amplification reaction was performed with 300 ng of genomic DNA extracted from hESCs, hiPSCs and hFFs. The primers used to amplify the transgene are presented in Supplementary Table 1.

*Bisulfite pyrosequencing*

One microgram of genomic DNA (per sample) isolated from hFFs, hESCs, and iPSCs was used for the bisulfite conversion and subsequent sequencing. The bisulfite conversion was performed using the EZ DNA Methylation Kit (Zymo Research) according to the manufacturer’s instructions. The promoter regions of Oct4 and Nanog were amplified by PCR and cloned into the pCR2.1-TOPO vector (Invitrogen). Eight to ten random clones were sequenced using the M13 forward and M13 reverse primers. The primer sequences used in the PCR amplification are shown in Supplementary Table 1.

*Teratoma formation*

One million OSKM-hiPSCs were harvested and injected subcutaneously into the dorsal flank of six-week-old SPF/VAF immunodeficient mice (Orientbio). Eight to twelve weeks after injection, visible tumors were dissected and fixed overnight with a 4% paraformaldehyde/PBS solution. Paraffin-embedded tissue was sectioned and stained with hematoxylin and eosin and examined for the presence of tissues representative of all three germ layers.

*Statistical Analysis*

All of the data are expressed as the mean ± SEM. Differences between the groups were analyzed using the Student’s t-test. A p-value ≤0.05 was considered significant. All of the data are representative of at least three independent experiments.
